# Adaptation biases the parallel perception of subitized numerosities

**DOI:** 10.1038/s41598-024-76536-1

**Published:** 2024-10-29

**Authors:** Wei Liu, Xiaoke Zhao, Ying Liu, Yating Li, Jingguang Li

**Affiliations:** 1 College of Education, Yunnan Minzu University, Kunming, China; 2 College of Teacher Education, Dali University, Dali, China

**Keywords:** Numerosity adaptation, Subitizing, Estimation, Parallel processing, Gist perception, Psychology, Cognitive neuroscience

## Abstract

Numerosity adaptation is a phenomenon in which prolonged exposure to a stimulus of greater numerosity makes subsequent stimuli appear less numerous, and vice versa. It has been confined to moderated numerosities outside the subitizing range (> 4). This study investigated whether the estimation of small numerosities (1–4), which is performed rapidly and accurately due to the mechanism of subitizing, is susceptible to adaptation. After adapting to a 50-dot stimulus, participants were presented with stimuli consisting of 1–5 color sets. In some trials, participants were informed of the target color-set before the presentation of stimuli, while in others, they were instructed afterwards. When estimating 1–4 dots in the single-color set or superset (the total dots), no adaptation effect was observed. The coefficient of variation (CV) was below 0.05, indicating the effective function of subitizing. However, when enumerating subsets in parallel, adaptation biased the estimation. The CV in estimating subitized numerosities was comparable to and correlated with that of estimating moderate numerosities (5–12), suggesting that subitizing was superseded by numerosity estimation. Greater effects arise when the targets were probed afterwards, with elevated CV. The prior adaptor may be more weighted to optimize detection of number deviations, especially under higher perceptual uncertainty.

## Introduction

Humans, as well as non-human animals, demonstrate a comprehension of non-symbolic numbers soon after birth^1–4^. Human infants are capable of discriminating between numbers differing in a 1:2 ratio^1^, and this ratio increases to 7:8 for adults, consistent with Weber’s Law^5^. However, there is ongoing debate about whether this discrimination relies on a direct sense of number or whether numerosity is indirectly derived from other congruent cues^6^. The notion that perception of numerosity is a directly sensed perceptual category is largely supported by the fact that numerosity exhibits adaptation^7^.

Numerosity adaptation refers to the phenomenon where the perception of numerosity decreases after adapting to a larger number of stimuli, and conversely, it increases after adapting to a smaller number^7–9^. Numerosity adaptation is independent of the processing of visual inputs, such as size, shape, color, or contrast of stimuli, and it occurs across different stimulus modalities and formats^10–12^. The substantial adaptation effects for the perception of quantity, occurring in the absence of adaptation effects for other features, were interpreted as evidence that the perception of numerosity behaved similarly to other primary visual properties (such as color, orientation or motion)^13^. Recent studies have pointed out that numerosity adaptation exhibits some traits that distinguish it from those of low-level covarying features. For example, numerosity adaptation effects can be induced by brief adaptation periods and are integrated with eye-movement information across cascades to form an isomorphic map in the visual cortex anchored in stable real-world coordinates^10,13,14^. In contrast, adaptation of low-level attributes such as density, contrast, or orientation relies on longer adaptation periods, and its effects primarily remain within the adapted retinal region^13–15^. However, it remains compelling to determine whether this adaptation operates through a mechanism dedicated to numerosity or through low-level texture-like mechanisms, as the perceived number cannot be isolated from all confounding magnitudes that are correlated with number^6,14,16–18^.

Number of a few items (up to four) can be appraised in a rapid and errorless manner based on a mechanism known as subitizing^19,20^. When the item number exceeds the subitizing range (yet remains within a moderate range), it is estimated with a reasonable error rate, relying on the numerosity mechanism^18,21,22^. Whilst being fast and accurate, subitizing is highly dependent on attentional resources^23,24^. Visual attention is efficient for selecting and enumerating the number (7–30 dots) of three sets in parallel: two color subsets and the superset, which is compatible with the three-item limits of object-based attention^25^. However, with very few items (1–4), subitizing only occurs in the single-set condition, where only one color-set is presented, and in the superset condition, where the total number of all dots is required. The error-free subitizing is absent in multiple-subset conditions, where the participants must distribute their attention and simultaneously enumerate more than one subset^21^. In other words, subitizing lacks the capability for processing subsets in parallel. In that case, estimation carries out the enumeration with a reasonable precision^21,22,26^.

Most studies of numerosity adaptation have been confined to the moderate numerosity range. Under normal conditions adaptation has little effect in the low subitizing range^16^. A previous study suggested that susceptibility to numerosity adaptor stimuli emerges at very low numerosities under conditions of attentional deprivation. When attentional load is induced by a dual-task paradigm, perception of the three-dot reference is biased against the adapted numerosity^18^. Even at low numerosities, the mechanisms of numerosity and subitizing may operate concurrently, and numerosity adaptation can affect subitized numerosities particularly when subitizing is disrupted by attentional load^18^.

The current study aimed to confirm and expand upon the previous study^18^ by investigating whether the perception of subitized numerosities (1–4) is susceptible to adaptation when attention is distributed across multiple subsets. The estimation paradigm was used to directly describe the perception of subitized numerosities, without resorting to testing series that might exceed the subitizing range. Participants were presented with stimuli comprising multiple subsets (from 1 to 5) defined by different colors, which changed on every trial. In some trials, participants were instructed about the target color before stimulus presentation (probe-before condition), while in other trials, they were instructed after stimulus presentation (probe-after condition). Once the target was cued, the participants had to estimate its numerosity. Prior to the testing stage, the adaptor stimulus was presented. The estimation of target number under the adapted condition was compared with that under the non-adapted condition, in order to determine the occurrence of adaptation. The coefficient of variation (CV) of estimation was computed in order to derive the processing precision in each condition.

Anticipating the results, we found that adaptation biases the perception of subitized numerosities when participants were required to enumerate more than one subset during the testing stage (i.e., when the target was cued after the presentation of multiple subsets). In this condition, CV of estimating subitized numerosities (1–4) was comparable to and correlated with that of estimating moderate numerosities (5–12)^21^, indicating that subitizing is superseded by numerosity estimation under attentional load. Moreover, the aftereffects of adaptation were significantly stronger in the probe-after condition compared to the probe-before condition, both for subitized and moderate numerosities. This suggests that numerosity adaptation is modulated by attention. Greater adaptation effects occur in accompaniment with higher CV, which reflects higher perceptual uncertainty induced by attention allocation. Rather than being a by-product of neural fatigue resulting from processing low-level degradation of inputs, numerosity adaptation reflects perceptual flexibility. In the case of higher uncertainty, the prior adaptor would be more weighted to optimize the detection of deviation in subsequent numerosity perception.

## Method

### Statement of ethical approval

For both of the experiments, the data were analyzed anonymously. All participants provided their informed consent according to the Declaration of Helsinki prior to the experiment in both verbal and written forms, and they were compensated for their participation. Yunnan Minzu University’s ethics committee approved this study(No.20230913-01).

## Sample size and participants

With an α error probability of 0.05 and a power of 0.8, power analysis using G*power 3.1 showed that fifteen participants were required for repeated measures ANOVA with a 0.4 effect size, and thirty participants were required for correlation analyses with a medium effect size (0.35). A total of thirty participants aged 17 to 32 years (averagely aged 24 years, 10 males) with normal or corrected-to-normal vision and normal color perception were recruited. A total of 720 trials were conducted for each participant. Specifically, 57% of the trials with target number ranges of 1–4, and 36% of the trials with target number ranges of 5–12 were analyzed separately in the subitizing and numerosity groups. Compared with previous studies, sufficient participants were enrolled, and sufficient trials were provided in each condition^21,25^. The remaining 6% of the trials, in which the target was not shown or the subset number exceeded three, were excluded from the analyses. In particular, the trials with four or five subsets were excluded due to their insufficiency for statistical analysis.

## Stimuli and procedure

**Fig. 1:**
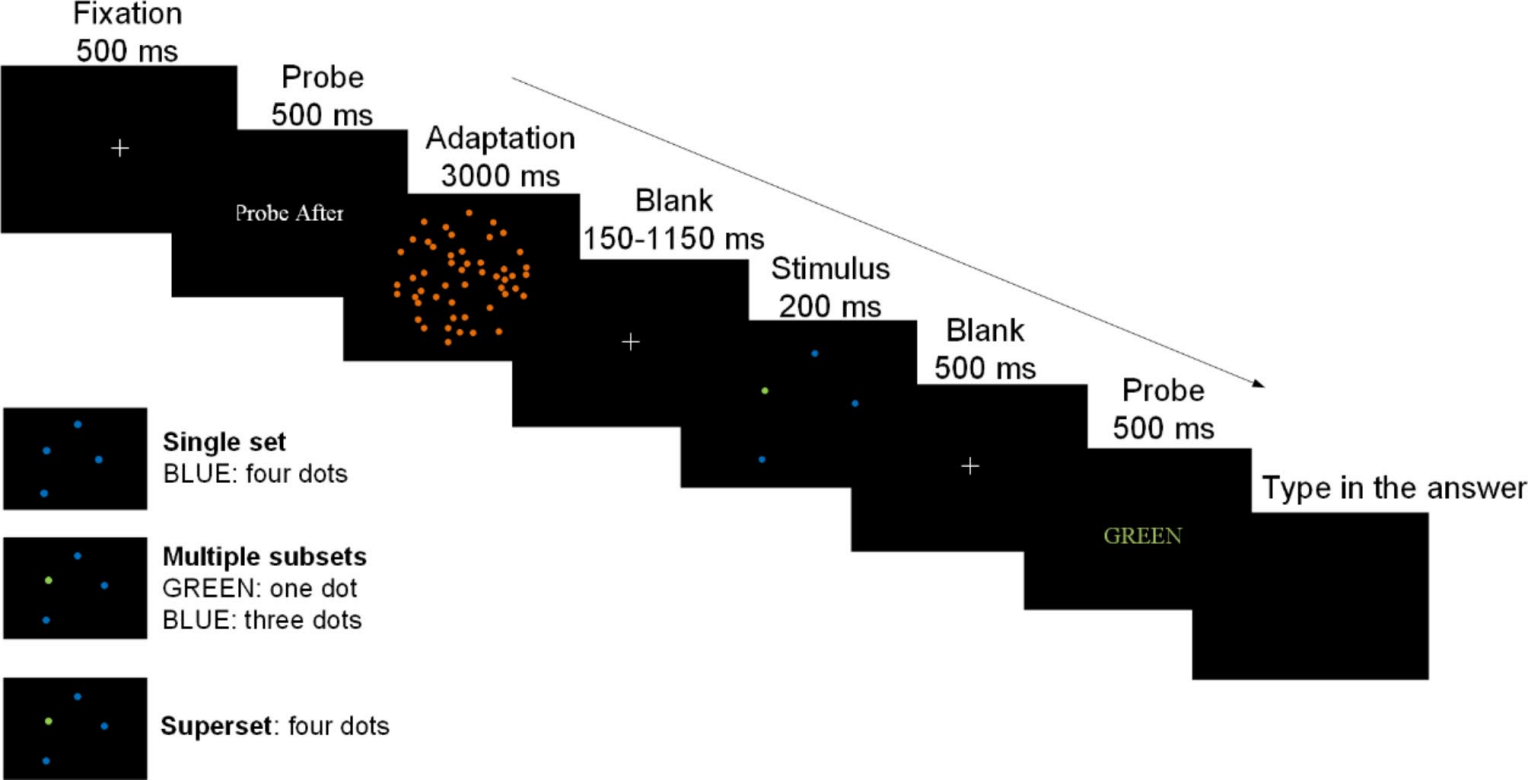
Schematic illustration describing the procedure of the adaptation conditions. The adaptation started with a fixation lasting for 500 ms, followed by a probe lasting for 500 ms, and an adaptor lasting for 3000 ms. In the “probe-before” condition, the target color-set was cued on the probe slide, whereas in the “probe-after” condition, this screen indicated ‘Probe After’. Then, it proceeded to the testing phase. At the beginning of each trial, a fixation was shown for 150–1150 ms. Then, participants saw a 200-ms stimulus display containing 1–12 dots of one to five colors. The stimulus was followed by a blank slide lasting for 500 ms. If the first probe screen stated ‘Probe After’, there would be a second probe slide stating the target for 500 ms, which followed the blank slide. The participants enumerated the number of the target, which could be one of the color subsets or the superset, typing their answers into the computer after the onset of the second probe. The correct responses for each probe condition are listed in the bottom left corner of Fig. 1.

Participants sat approximately 50 cm from an LCD monitor with a viewable area measuring 41 cm by 26 cm (19”, 1920 × 1080, 60 Hz) in a dimly lit quiet room. Stimuli were generated from Matlab (Mathworks, Natwick, MA). The diameter of each dot is 0.5° visual angle (20 pixel). Dots were presented in a circle at the center of the screen with a diameter of 15° (600 pixel) visual angle. Figure 1 illustrates the procedure for the adapted conditions. The adaptation began with a fixation lasting for 500 ms, followed by a probe lasting for 500 ms, which indicated the target color in the probe-before condition, or indicated ‘Probe After’ in the probe-after condition. Then, an adaptor showed up. It was presented for 30 s on the first trial and appeared for 3000 ms at the beginning of each subsequent trial. The participants should keep their eyes on the central adaptor. Next, it proceeded to the testing phase. The paradigm of the testing phase followed that of previous studies^21,25^. At the beginning of each trial, a fixation was displayed for 150–1150 ms. Subsequently, participants were presented with a 200-ms stimulus display containing 1–12 dots of one to five colors. The stimulus was followed by a blank slide lasting for 500 ms. If the first probe screen stated ‘Probe After’, it would be followed by a second probe slide displaying the target color for 500 ms, which succeeded the blank slide. Else if the first probe screen stated the target color, no probe would be presented. The participants were required to enumerate the number of dots in the target, which could be one of the color subsets or the superset, and they entered their answers into the computer following the appearance of the second probe.

Dots in stimulus patch were randomly distributed with the constraint that they could not overlap with each other. The number of dots in each color subset was randomly determined, and in half (49.6%) of the trials the target subset was smaller than at least one distracting subset, making the strategy of attending only to the largest subset ineffective. For each participant, an average of 360 trials which combined all conditions in a randomized order were run in 3 blocks. Generally, on 22% of the trials, participants were asked to report the overall number of the dots regardless of the colors (superset trials). The proportion of one-, two-, and three-color subset conditions was 19%, 32%, and 21%, respectively. In each of the subset/superset group, the proportion and target number range of the probe-before and probe-after conditions are approximately equal. In 2% of the trials, there were four or five color subsets. In 4% of the trials, there was no target color represented, and the correct respond is ‘0’. Proportion of each condition is slightly different among participants.

The procedure in the adapted condition was similar to the baseline condition except that there was an adaptation stage prior to the testing procedure. Adaptor always comprised 50 orange dots, which was different from the color of testing dots (blue, green, Magenta, red, or yellow).

The participants were asked to keep their eyes on the middle fixation during the whole trial. The baseline condition was conducted prior to the adapted condition for each participant. Adequate rest was ensured to avoid fatigue.

### Data analysis

As there were various target numbers, a normalized bias was calculated as the ratio between the average response and target numerosity for each numerosity. This ratio was averaged across all target numerosities separately for each condition to assess accuracy. Then, the adaptation effects *(A)* were calculated by the difference in the averaged ratio between baseline and adaptation conditions,

1 $$A=R_{Base}-R_{Adapt}$$

where *R*_Base_ and *R*_Adapt_ refer, respectively, to the averaged ratio in the baseline and adaptation conditions. These adaptation effects were compared to ‘0’ using a one-sample *t*-test to determine whether the adaptation induced significant underestimation. The coefficient of variation (CV) was calculated as the ratio between the standard deviation (SD) and the mean of enumeration. It provides a dimensionless measure of the noise in the judgments, allowing comparison of performance precision across numerosities, as well as comparison between baseline and adapted (biased) conditions^21,24^. Pearson correlation coefficient *r* was calculated for CV between related conditions. In this study, *t*-tests are two-tailed. Cohen’s *d* was reported to provide a complement to null hypothesis statistical significance testing by estimating the magnitude of the difference (0.2–0.5 for small effect size, 0.5–0.8 for moderate effect size, and > 0.8 for large effect size). Bayes factors (*BF*_10_) were reported to estimate whether the null hypothesis H_0_ or the alternative hypothesis H_1_ is more likely to be correct. *BF*_10_ < 0.3 suggests clear evidence for H_0_, whereas *BF*_10_ > 3 indicates clear evidence for H_1_.The False Discovery Rate probability (FDR), or *Q* value, is adopted in this study to correct probability of type- I error in multiple comparisons^27^. For example, with four *p* values in multiple comparisons, we multiply the smallest *p*-value by four to get its *Q* value. Then we multiply the second smallest *p* by 4/2, the third *p* by 4/3, and the last *p* (the largest one) by 4/4 to get their *Q* values. To determine whether a comparison is statistically significant, *Q* values are used instead of *p* values, *Q < 0.05, **Q < 0.01, ***Q < 0.001. *Q* = 0.05 is a widely accepted threshold for significance.

## Results

### Adaptation effects

**Fig. 2:**
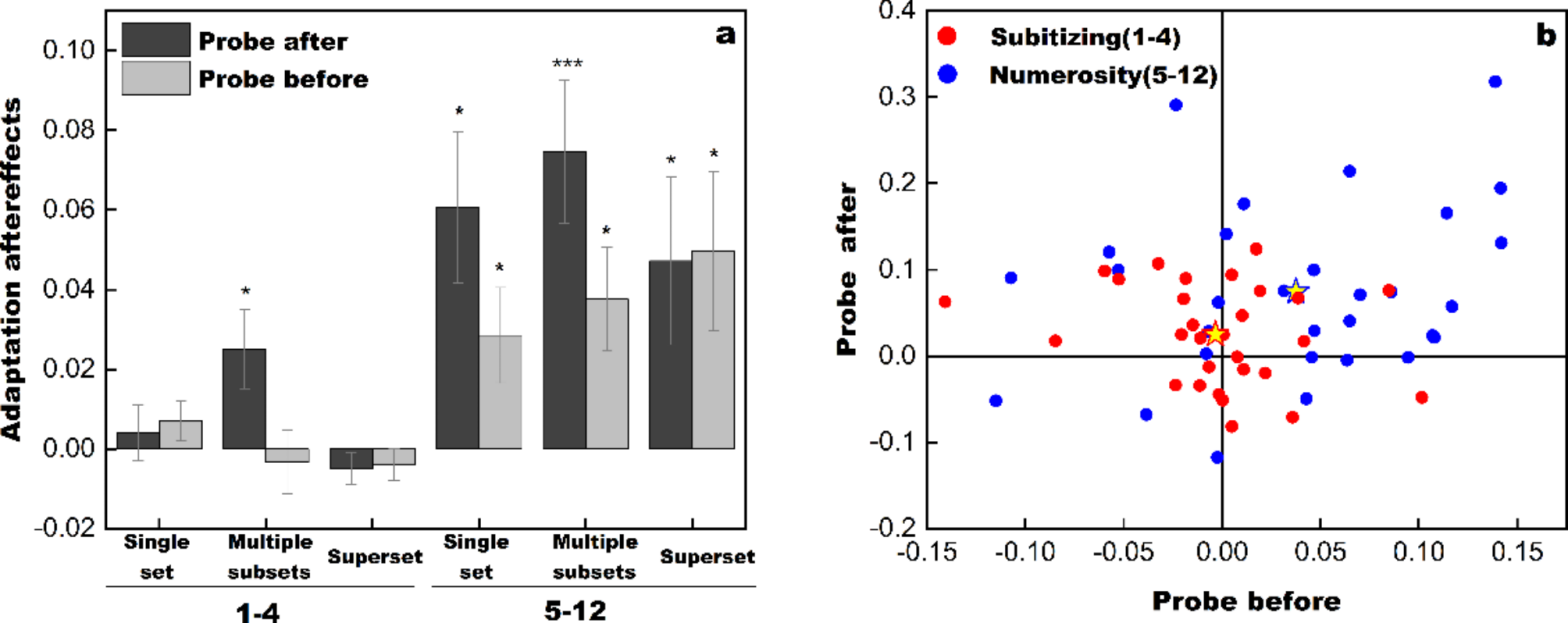
Results of adaptation effects. The adaptation effects are assessed by subtracting the mean estimation ratio between the baseline and adaptation conditions. (a) Three clusters on the left demonstrate the results within the subitizing range (1–4). Significant underestimation is induced by adaptation in the probe-after condition with multiple color subsets. No adaptation effects are observed in other conditions. Three clusters on the right show the results within the numerosity range (5–12). Adaptation effects are significant in each condition. When multiple color subsets were simultaneously enumerated in the probe-after condition, the adaptation effect is greater, both for the subitizing and numerosity groups. Error bars represent one standard error. Asterisks indicate the significant level, *Q < 0.05, **Q < 0.01, ***Q < 0.001. (b) Individual adaptation effects in probe-before and probe-after conditions with multiple color subsets. Red dots represent for the aftereffect of subitizing targets, and blue dots represent for numerosity targets. Starts denote the mean of each group.

Figure 2a displays the adaptation effects in different conditions. As the adaptation effects are assessed by subtracting the mean estimation ratio between the baseline and adaptation conditions, a positive value indicates the presence of adaptation effects, i.e., underestimation induced by the adaptor.

### Subitizing group (1–4)

For target numbers within subitizing range (1–4, three clusters on the left of Fig. 2a), a repeated-measures ANOVA was conducted with adaptation effects as the dependent variable. This model returned no significant main effect of probe condition (after/before), *F*(1, 29) = 1.635, *p* = 0.211, η_p_^2^ = 0.053, *BF*_10_ = 0.455, a marginal significant main effect of target set (one/multiple/superset), *F*(2, 58) = 3.113, *p* = 0.052, η_p_^2^ = 0.097, *BF*_10_ = 0.575. Importantly, a significant interaction was found, *F*(2, 58) = 3.329, *p* = 0.043, η_p_^2^ = 0.103, *BF*_10_ = 1.809. Simple main effect tests indicated a significant difference among different target sets in the probe-after condition, *p* = 0.014. After adaptation, the targets presented within multiple subsets were significantly underestimated in comparison to the other types of target sets. No difference was suggested in the probe-before condition, *p* = 0.334.

Then, we analyzed whether the adaptation effects varied significantly from zero in each condition. Significant underestimation was observed in the probe-after condition with multiple color subsets (2–3 color groups), *t*(29) = 2.407, *p* = 0.023, *Q* = 0.053, Cohen’s *d* = 0.439, *BF*_10_ = 2.270, indicating that numbers within the subitizing range are biased by adaptation when attention is distributed across subsets, as the target is cued after the stimuli. No bias was observed in the probe-before condition with multiple subsets, *t*(29) = -0.402, *p* = 0.691, *Q* = 0.691, Cohen’s *d* = -0.073, *BF*_10_ = 0.210.

No bias was observed under any other condition, whether in the one-set or superset scenario, irrespective of whether the target was probed prior to or subsequent to the presentation of the stimuli slide, *p * ≥0.168, *Q* ≥ 0.252, *BF*_10_ ≤ 0.500.

In the subitizing group, it’s worth noting the target numbers in all conditions fell within the subitizing range (1–4), even for the superset condition.The trials involving more than four target-dots were analyzed in the numerosity group (5–12). The data was subdivided in this manner to enable us to compare different conditions within the same numerosity regime. In the probe-before conditions, enumeration was focused on the target set. In the probe-after conditions, item numbers within the multiple color subsets (as well as within the superset) were perceived in parallel, i.e., the dot numbers of both the subsets and the superset were processed simultaneously. The perceived number of the target set was reported when the color subset or the superset was cued after the stimuli^21,25^. Interestingly, when the participants were asked to enumerate the total number of items in the superset, they consistently perceived it in an unbiased manner, even when the target was probed subsequent to the testing stimuli, and even when the superset contained up to five color subsets. The priority of processing the superset will be elucidated in the discussion.

### Numerosity group (5–12)

Three clusters of bars on the right of Fig. 2a demonstrate the results within the numerosity range (5–12). Adaptation effects are significant in each condition, *p ≤* 0.032, *Q* ≤ 0.055, *BF*_10_ ≥ 1.732. The repeated-measures ANOVA with adaptation effects as the dependent variable returned no main effect of probe condition (after/before), *F*(1, 29) = 3.343, *p* = 0.078, η_p_^2^ = 0.103, *BF*_10_ = 0.838, and no main effect of target set (one/multiple/superset), *F*(2, 58) = 0.324, *p* = 0.724, η_p_^2^ = 0.011, *BF*_10_ = 0.075. No interaction was found, either, *F*(2, 58) = 1.133, *p* = 0.329, η_p_^2^ = 0.038, *BF*_10_ = 0.226. For multiple-subset targets, simple main effect tests indicated a marginal significant difference between probe conditions, *p* = 0.067. Targets within multiple subsets tended to be further underestimated when they were cued after the stimuli. No difference was found in other conditions.

### Individual data

To better illuminate the adaptation effects in the multiple-subset conditions, the individual data in the probe-before and probe-after conditions are shown in Fig. 2b. As for targets within the subitizing range (1–4, red dots), underestimation primarily occurs in the probe-after condition, rather than the probe-before condition, for most participants. By contrast, adaptation biases numerosity perception both in the probe-after and probe-before conditions for targets within the numerosity range (5–12, blue dots).

## The coefficient of variation (CV)

### Subitizing group (1–4)

**Fig. 3:**
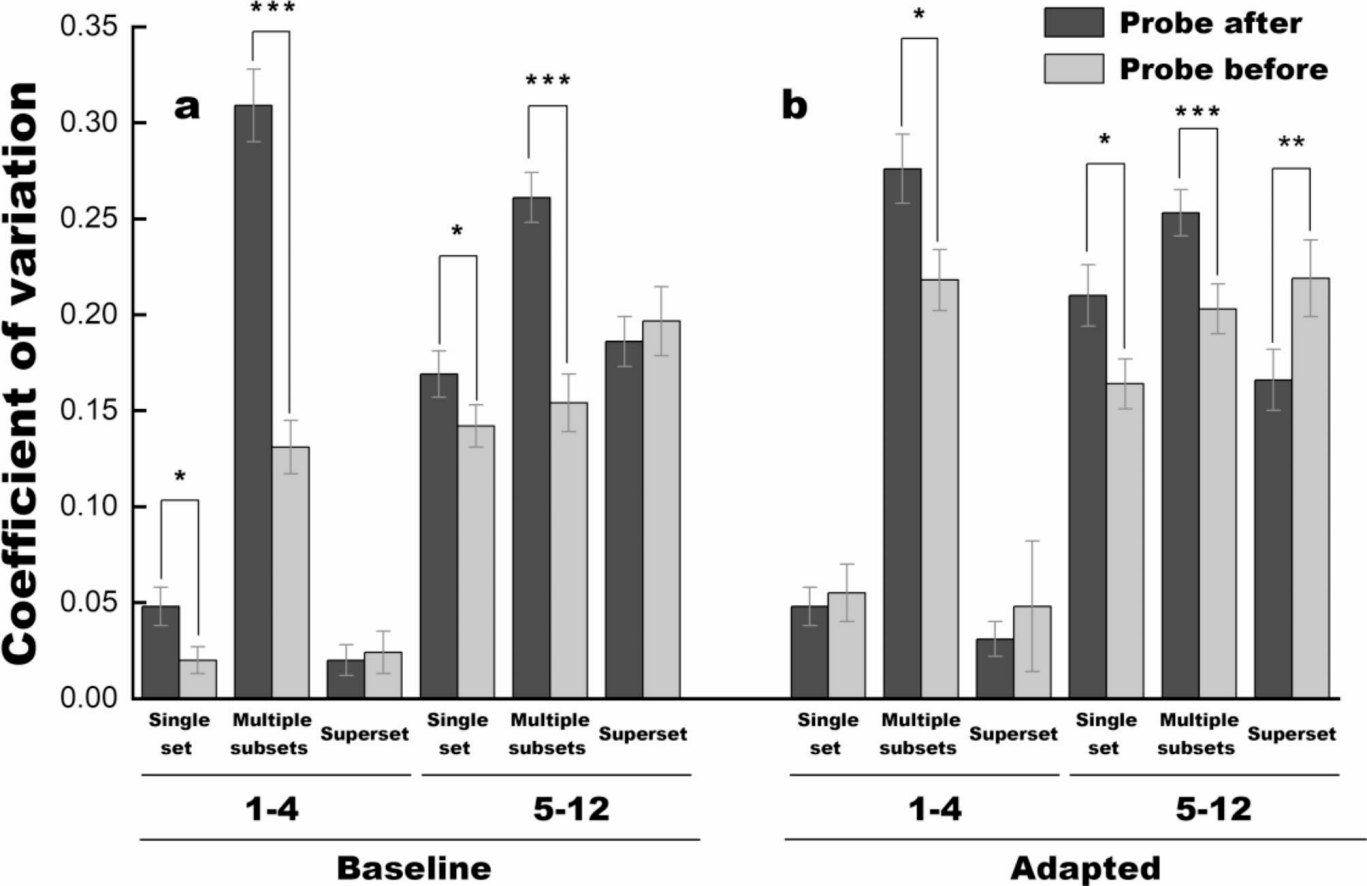
Coefficient of variation for estimation ratio. CV provides a dimensionless measure of the noise in judgments, which allows comparison of performance precision across numerosities, as well as comparison between baseline and adaptation (biased) conditions. (a) Results in the baseline condition. For numbers within the subitizing range (1–4), CV for enumerating a single set is below 0.05, highlighting the hallmark of errorless subitizing. When multiple color subsets are presented, CV reaches 0.13–0.31, even when the target subset is probed before stimulus onset. The signature of approximate estimation is evident in these conditions. Noticeably, CV falls back to near zero when the superset is asked, regardless of whether the target number is probed before or after the stimulus, suggesting an activation of subitizing. For numbers beyond the subitizing range (5–12), CV is above 0.14, indicating the activity of an approximate numerosity mechanism. Both for subitizing and numerosity groups, CV for enumerating multiple subsets is higher in the probe-after condition, compared to the probe-before condition. By contrast, no such difference is observed in the superset groups. (b) Results in the adaptation condition. The CV pattern is reminiscent of that in the baseline condition. Error bars denote one standard error. Asterisks indicate the significant level, * p  < 0.05, ** *p*  < 0.01, *** *p*  < 0.001.

Figure 3a displays CV in baseline conditions. As for the subitized range of 1–4, CV in single-set conditions is below 0.05. These results are consistent with the idea that subitizing is engaged and performance is almost error-free. In the multiple-subset group, however, CV raises considerably and ranges between 0.13 and 0.31 across conditions. It is clear that errorless subitizing is absent. When there are two or three subsets in the visual field, participants cannot subitize them even when they are told in advance which one is the target. In superset condition, notably, CV drops below 0.03, both on probe before and after trials. Thus, participants can subitize if they are asked to enumerate all dots, irrespective of probe conditions and the colors defining each group.

The repeated-measures ANOVA with CV as the dependent variable returned a significant main effect of probe condition, *F*(1, 29) = 49.810, *p* < 0.001, η_p_^2^ = 0.632, *BF*_10_ > 100, a significant main effect of target set, *F*(2, 58) = 148.895, *p* < 0.001, η_p_^2^ = 0.837, *BF*_10_ > 100, and a significant interaction, *F*(2, 58) = 38.025, *p* < 0.001, η_p_^2^ = 0.567, *BF*_10_ > 100. According to the simple main effect tests, for the superset targets, no significant difference was observed between the probe conditions, *p* = 0.672. However, the CV in the probe-after condition was significantly higher than that in the probe-before condition, both for the one-set targets, *p* = 0.010, and for the multiple-subset targets, *p* < 0.001.

### Numerosity group (5–12)

As for the numerosities of 5–12, CV range between 0.14 and 0.27 across conditions. The repeated-measures ANOVA returned a significant main effect of probe condition, *F*(1, 29) = 18.490, *p* < 0.001, η_p_^2^ = 0.389, *BF*_10_ > 100, a significant main effect of target set, *F*(2, 58) = 9.446, *p* < 0.001, η_p_^2^ = 0.246, *BF*_10_ = 7.130, and a significant interaction, *F*(2, 58) = 13.694, *p* < 0.001, η_p_^2^ = 0.321, *BF*_10_ > 100. According to the simple main effect tests, for the superset targets, no significant difference was observed between the probe conditions, *p* = 0.541. However, the CV in the probe-after condition was significantly higher than that in the probe-before condition, both for the one-set targets, *p* = 0.039, and for the multiple-subset targets, *p* < 0.001. The performance pattern is reminiscent of the data pattern within the subitizing numerosity range.

Previous studies suggested that we can estimate up to three sets in parallel without a tangible cost: two subsets and superset^21,25^. In this study, we have combined the two- and three-subset conditions to get a stable data for analyses, hence CV is larger in the probe-after multiple-subset condition. Nevertheless, in line with previous studies, our data shows that participants can enumerate superset effectively, irrespective of probe conditions and the colors defining each group.

Figure 3 illustrates the CV in the adaptation conditions. (1) Within the subitizing range, ANOVA returned no effect of probe condition, *F*(1, 29) = 0.533, *p* = 0.471, η_p_^2^ = 0.018, *BF*_10_ = 0.209. There was a significant effect of target set, *F*(2, 58) = 102.335, *p* < 0.001, η_p_^2^ = 0.779, *BF*_10_ > 100. No interaction was observed, *F*(2, 58) = 2.090, *p* = 0.133, η_p_^2^ = 0.067, *BF*_10_ = 0.754. Simple main effect tests showed no difference between the probe conditions, neither for the superset targets, *p* = 0.628, nor for the one-set targets, *p* = 0.668. For the multiple-subset targets, however, the CV in the probe-after condition was significantly higher than the probe-before condition, *p =* 0.032. (2) Within the numerosity group, ANOVA yield no effect of probe condition, *F*(1, 29) = 3.539, *p* = 0.070, η_p_^2^ = 0.109, *BF*_10_ = 0.314. There is a significant effect of target set, *F*(2, 58) = 3.418, *p =* 0.040, η_p_^2^ = 0.105, *BF*_10_ = 3.268. A significant interaction was also observed, *F*(2, 58) = 11.323, *p* < 0.001, η_p_^2^ = 0.281, *BF*_10_ > 100. When the targets were cued after the stimuli, CV increased for the one-set targets, *p* = 0.017, and for the multiple-subset targets, *p* < 0.001. By contrast, CV for enumerating superset does not increase when the target is probed afterwards. It even decreases, *p =* 0.004. To conclude, when the target was cued afterwards, CV did not increase when the superset was queried, whereas it increased in the face of subsets.

### CV correlation

**Fig. 4:**
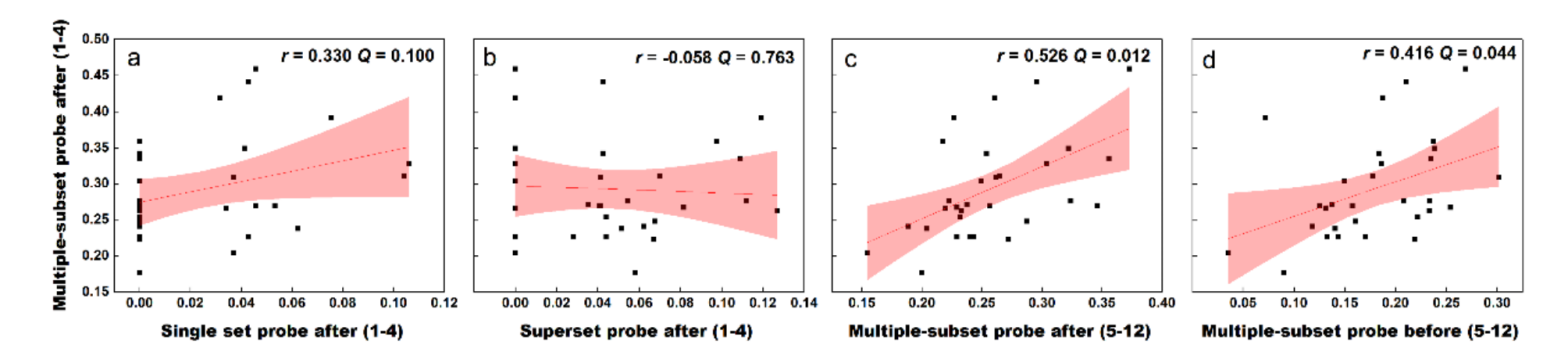
Scatters for individual CV. When target numbers are within the subitizing range (1–4), a significant adaptation effect was observed when there were multiple subsets, and when the targets were cued after stimuli. To check if this adaptation reflect the activity of the numerosity system, we investigate the CV correlation of this condition across other conditions. Within the subitizing range, no significant CV correlation is found, either between this condition and the single-set condition (a), or between this condition and the superset condition (b), even if the target number range was identical, and the targets were probed in the same way (after the stimuli). By contrast, with multiple subsets, CV in this subitizing condition is significantly correlated with that in the numerosity group (5–12), despite the targets being probed before (c) or after (d) the stimuli. Pink area donates 95% CI.

When target numbers are within the subitizing range, a significant adaptation effect was observed when there were multiple subsets, and the targets were cued after stimuli (Fig. 2a). In addition, CV of this condition was revealed to be comparable with that in the numerosity group. To check if the results in this condition reflect a genuine result of the numerosity system, we investigated the CV correlation of this condition across other conditions. We first calculated the CV correlation across the probe-after conditions within the subitizing group. No significant CV correlation is found between this multiple-subset condition and the single-set condition (Fig. 4a), *r* = 0.330, *p* = 0.075, *Q* = 0.100, *BF*_10_ = 1.033, and no significant correlation was found between this multiple-subset condition and the superset condition (Fig. 4b), either, *r* = -0.058, *p* = 0.763, *Q* = 0.763, *BF*_10_ = 0.237. Then we analyzed the CV correlation across the multiple-color conditions between subitizing and numerosity groups. Significant CV correlation was observed between this subitizing and the numerosity conditions: *r* = 0.526, *p* = 0.003, *Q* = 0.012, *BF*_10_ = 16.041 for the numerosity condition in which the target was cued after the stimuli (Fig. 4c), and *r* = 0.416, *p* = 0.022, *Q* = 0.044, *BF*_10_ = 2.739 for the condition in which the target was cued before the stimuli (Fig. 4d).

To sum up, in the face of multiple subsets, perception of subitized numerosities should be based on numerosity mechanisms. This point of view is supported by two evidences. First, the perception is susceptible to adaptation. Second, the processing precision is comparable to and significantly correlated with that for moderate numerosities.

## Discussion

Under normal condition, numerosity adaptation is confined to moderate numerosities^7,18,27,28^. Debate exists in whether numerosity adaptation is based on a mechanism dedicated to numerosity or via texture-like mechanisms^6,16,17,29^. The current study investigated whether the perception of subitized numerosities (1–4) is susceptible to adaptation when attention is distributed across multiple subsets. The result that subitized numerosities are also susceptible to numerosity adaptation contributes in elucidating the mentioned debate, as a mechanism based on extensive measures could hardly operate at such low numerosities. In addition, the current study detected a robust adaptation effect in central vision, both for the subitizing and numerosity regimes. Previous studies have suggested that texture mechanisms may supersede numerosity when individual items become indistinguishable^30,31^. As the transition point from numerosity to density is dependent on eccentricity (i.e., a crowding-like effect)^31^, it is possible that adaptation in peripheral vision is partly a consequence of the processing of continuous textures. Nevertheless, in central vision, it is evident that the perception and adaptation of numerosity operate directly on segregate objects.

A series of studies have suggested that even at subitized numerosities, the mechanisms of numerosity and subitizing may operate concurrently, and numerosity will take over the job of enumeration when subitizing cannot benefit from attention. In that case, similar processing features can be observed both for subitized and moderate numerosities^18,21,22,26^. This study went further to investigate whether adaptation biases the perception of subitized numerosities, as it does with moderate numerosities, when attention is distributed across multiple subsets. The current findings indicate that, although accuracy remains unchanged in the single-set scenario, suggesting that the perception of subitized numerosities remains unaffected by adaptation when subitizing occurs under normal conditions, it becomes evident that adaptation does influence this perception when participants are required to enumerate multiple subsets concurrently during the testing phase.

Our previous study^21^ have pointed out that subitizing lacks the capability to process subsets in parallel. With multiple subsets, when the target was probed after the stimuli, participants had to simultaneously enumerate the numerosity of each subset. Errorless subitizing disappeared as it cannot benefit from distributed attention. In the current study, compared to the single-set condition, the CV of estimating subitized numerosities in the multiple-subset condition increases from 3 to 31%, which is comparable with the CV of estimating moderate numerosities (5–12) in the same condition (27%). In addition, the CV of estimating subitized numerosities in this multiple-subset condition is positively correlated with that of estimating moderate numerosities in the same condition, whereas it is not correlated with the CV in the single-set or superset conditions, despite the identical target number range across these conditions. These results are in line with our previous study, indicating that while subitizing functions effectively in processing single set and superset (mean CV is below 3%), it is superseded by numerosity estimation under the attentional load induced by processing multiple subsets^21^. Furthermore, this study offers novel evidence indicating that adaptation occurs in the multiple-subset group with the probe-after-target paradigm, yet it is absent in the single-set or superset groups, regardless of the probe conditions. In other words, adaptation may occur when numerosity takes over the job of enumerating small numerosities^18^.

Subitizing performs well in the superset condition, irrespective of whether the target is cued before or after, suggesting that the processing of superset possesses a certain degree of invulnerability to the availability of attentional resources^21^. Consistent with our previous research, the current results indicate that adaptation cannot bias the perception of subitized numerosities when enumerating “all” numbers. Regardless of the probe conditions, both the precision and the accuracy of the perception remains unaffected. It’s worth mentioning that a total of no more than four dots were present under the superset condition. One might be concerned that, in the face of a superset containing 1–4 dots, it would be simpler to infer “all numbers” by enumerating the subsets, each containing 1–2 dots respectively, and then summing them. However, further analysis reveals a CV of 0.20 in perceiving targets within the subsets, even when the total number (the superset) did not exceed four. In other words, even with 1–4 dots in total, accurate subitizing only persists when the superset is queried. The priority of processing superset has also been illuminated in the moderate group: both the adaptation effects and the CV remain constant irrespective of the probe conditions. These results provide further evidence supporting the idea that the superset is processed by an efficient perceptual subsystem^32^.

According to our previous research, the simple request to segregate the visual scene into two groups can drain resources critical for subitizing. When distinct color subsets are presented simultaneously, subitizing can be lost even when the target is cued before the stimuli^21^. In the current study, the CV (13%) indicates that subitizing is affected in the multiple-subset condition even when the target is probed before the stimuli. Given that the CV is substantially lower than that in the probe-after condition (31%), it is possible that subitizing can occasionally benefit from attention in this condition. When the target subset is notified prior to the presentation of the intermingled display, subitizing may be preserved with higher noise^33^, but it can also be superseded by numerosity when the non-target subsets are perceptually competitive enough to distract attention^21,22^. However, the noisy subitizing hypothesis cannot fully explain the absence of adaptation in this probe-before condition. We propose that attention enhancement may account for this lack of adaptation.

Previous study suggests that attention can enhance both the precision and the accuracy of numerosity perception, with a greater improvement in the enumeration of subitized numerosities and a weaker improvement for moderate numerosities^34^. Recent studies further point out that numerosity adaptation is also modulated by attention allocation in testing stages. In the testing stage, items in the cued patch can be overestimated compared to the opposite patch, and adaptation amplifies this cue-dependent numerosity bias^35^ (also in the current study). Numerosity adaptation reflects a mixture of both perceptual and attentional processes^35^, which may facilitate the subsequent perception of numerosity.

Adaptation had long been considered a by-product of neuronal activation fatigue. However, a series of studies have suggested that adaptation, especially to high-level attributes, may reflect a dynamic adjustment to improve our sensitivity to changes along the adapted dimension^13,35^. The repulsive bias caused by adaptation^36^ enhances the signal-to-noise representation, which therefore optimizes the detection of deviation along the adapted dimension in the subsequent perception. Assuming that adaptation serves to auto-calibrate perceptual systems to their environment, we can expect a greater perceptual bias when perception in the testing stage is marked by higher perceptual uncertainty. Taking CV as an assessment of perceptual uncertainty, the findings of the current study align with this hypothesis. Adaptation bars (Fig. 2a) and CV bars (Fig. 3, a&b) share a similar pattern, suggesting greater adaptation effects in conditions with higher CV: Greatest adaptation effects are revealed in multiple-after conditions in both number groups, along with the highest CV. For targets 5–12, similarly to that for targets 1–4, the adaptation effects in the probe-before condition are smaller than those in the probe-after condition, where higher uncertainty is induced by attention allocation. In the one-set and superset conditions of the subitized group, where the close-to-zero CV indicates high reliability in perceiving subitized target numbers, adaptation barely biases perception during the testing phase.

There are models linking adaptation effects to Bayesian prediction, suggesting that prior adaptor may serve as a standard for self-calibration^36,37^. In the Bayesian frame work, in accordance with the auto-calibration hypothesis, the effect of the prior depends on the relative reliability, and a greater adaptation effect is predicted under conditions of higher perceptual uncertainty^27,32^. It would be interesting to investigate this issue by modeling within the Bayesian framework in future studies.

## Data Availability

The data sets generated and analyzed during the current study are available from the corresponding author on reasonable request.
